# Effects of salt stress on antioxidant defense system in the root of *Kandelia candel*

**DOI:** 10.1186/s40529-014-0057-3

**Published:** 2014-07-23

**Authors:** He-ming Wang, Xiao-rong Xiao, Meng-ying Yang, Zhi-liang Gao, Jian Zang, Xiu-mei Fu, Yin-hua Chen

**Affiliations:** 1 Hainan Key Laboratory for Sustainable Utilization of Tropical Bioresource, Hainan University, Haikou, 570228 China; 2 College of Agriculture, Hainan University, Haikou, 570228 P. R. China

**Keywords:** Salt stress, Mangrove, Kandelia candel, Antioxidant defense system

## Abstract

**Background:**

This study aimed to explore the active oxygen scavenging mechanism of *Kandelia candel*, in order to provide a theoretical basis for further analysis on the physiological mechanism of salt tolerance in mangrove plants. Different concentrations of NaCl solution (0, 150, 300 and 450 mmol/L) were used for salt stress treatments on *Kandelia candel*, physiological indicators in the root of *Kandelia candel* were measured in different processing time.

**Results:**

With the increase of salt concentrations and processing time, the contents of total proteins in the root of *Kandelia candel* were reduced; the CAT activity, SOD activity, ASA content and MDA content all had decreased with the increase of salt concentrations and shown a trend from ascent to descent with the increase of processing time, the peak of ASA and MDA contents were observed at 6 h, that of SOD activity was observed at 9 h and that of CAT activity was at 12 h; POD activity had shown an overall upward trend with the increase of salt concentrations and processing time, which reached the maximum at 24 h; the variations of these physiological indicators were more significant in high concentrations of NaCl solution (450 mmol/L).

**Conclusions:**

A certain salt concentration (<300 mmol/L) was required for the growth of *Kandelia candel* seedlings. At the early stage of high-salt stress, *Kandelia candel* can rapidly activate antioxidant defense system to resist the salt induced oxidative stress, thus reducing the damages of oxidative stress to plasma membrane, which might be an effective means for *Kandelia candel* to resist high salt stress.

**Electronic supplementary material:**

The online version of this article (doi:10.1186/s40529-014-0057-3) contains supplementary material, which is available to authorized users.

## Background

Soil salinity is a major environmental factor affecting growth and development of plants. High salt leads to the reduction of yield or even death. Currently, salinization has been exacerbated in about 20% of agricultural land in the world, and over 50% of arable land will be saline and alkali by 2050 according to the prediction (Vinocur and Altman [2005]). A plant damaged by high salinity may suffer reduced shoot and root growth, yield losses and eventual death. These changes in plant growth are the result of salt’s detrimental effects on plant physiology which include ion toxicity, osmotic stress, nutrient deficiency and oxidative stress (Xiong and Zhu [2002]).

Oxidative stress is, in fact, a secondary effect of salinity, which causes the formation of reactive oxygen species (ROS), such as the superoxide anion (O2^•-^), hydrogen peroxide (H_2_O_2_), the hydroxyl radical (•OH) and singlet oxygen (^1^O_2_). ROS are produced and effectively neutralized during normal aerobic metabolism. ROS production increases to dangerous levels, which can damage proteins, lipids and nucleic acids by oxidation (Halliwell and Guteridge [1985]). In order to overcome oxidative stress, plants have developed two main antioxidants defense mechanisms that can be classified as non-enzymatic and enzymatic systems (Cheruth et al. [2009]). The first class (non-enzymatic) consists of small molecules such as vitamin (A, C and E), glutathione, carotenoids and phenolics which can react directly with the ROS by scavenging them. Second class is represented by enzymes, for example, superoxide dismutase, peroxidase and catalase which have the capacity to eliminate superoxide and hydrogen peroxide. Among them, superoxide dismutase catalyzes the first step of the enzymatic defense mechanism, the conversion of superoxide anions to hydrogen peroxide and water. If superoxide anions are not neutralized, oxidation occurs and hydroxyl radicals are formed. Hydrogen peroxide can be decomposed by the activity of catalases and several classes of peroxidases which act as important antioxidants (Frary et al. [2010]). The ability of certain species to increase production of antioxidant compounds and enzymes in response to salinity has been correlated with salt tolerance (Zhu et al. [2005]; Lopez et al. [1996]; Shalata et al. [2001]). Various studies have also shown that genetically engineered plants containing higher levels of ROS scavenging enzymes, such as SOD, APX, and POX, have improved tolerance to abiotic stresses such as salinity (Alscher et al. [2002]; Wang et al. [1999]; Roxas et al. [2000]).

Mangroves are a special kind of forest occurring in the intertidal zones of tropical and subtropical coastlines. They grew under conditions of high temperature, high radiation, high salt, low nutrition and long-term hypoxia, which have generated a set of effective active oxygen scavenging mechanism under long-term natural selection. *Kandelia candel*, a dominant species of mangrove plants in China, is a typical true mangrove species that can form rich stands in fields with salinities up to seawater level (~500 mnol/L NaCl) (Parida et al. [2004]). In this study, The SOD, CAT and POD activities and contents of soluble protein, ASA and MDA were measured in the seedling roots of *Kandelia candel* treated with different concentrations of salt for different processing time. The results may provide a theoretical basis for further analysis on the physiological mechanism of salt tolerance in mangrove plants.

## Methods

### Experimental design

Mature hypocotyls of well-developed *Kandelia candel* with no pests or diseases and with closely developmental maturity, length and weight (collected from Dongzhaigang Mangrove Nature Reserve in Hainan) were cultured with Hoagland nutrient solution in greenhouse. When the second pair of leaves were generated and began budding, the seedlings were transferred into NaCl solution with different concentrations for cultivation, including 150 mmol/L, 300 mmol/L and 450 mmol/L, respectively. Solution contained 0 mmol/L of NaCl was used as the control. Four pots with the same size were filled with 500 mL of Hoagland nutrient solution containing different concentrations of NaCl and 50 seedlings were planted in every pot and the processing time was set to 3 h, 6 h, 9 h, 12 h and 24 h, respectively. Three replications were set for each treatment and 3 seedlings in each replication. The roots for assay were freshly sampled and immediately subjected to enzyme assay after processing.

### Determination method

The content of total proteins was determined by using the coomassie brilliant blue G-250 staining method proposed by Bradford (Bradford, [1976]), the activity of SOD was determined using iodometric method, the activity of POD was determined via guaiacol colorimetric method, the activity of SOD was determined through the methods proposed by Lin (Lin et al. [2004]), the content of MDA was determined by spectrophotometric method and the content of ASA was determined using titration method (Parida et al. [2004]).

## Results

### Effects of salt stress on the content of total soluble proteins

**Figure 1:**
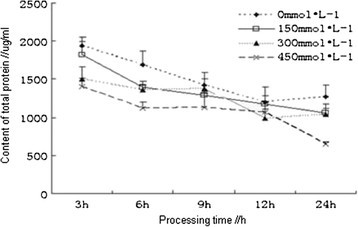
**Effects of salt stress on the contents of total soluble protein in the root of** ***Kandelia candel.***

Salt stress could result in variations in the number of proteins, inhibit the synthesis of most proteins and cause the decomposition of proteins. However, synthesis of some proteins was not affected by salt stress or even was enhanced. Therefore, the content of total proteins could not only be an indicator of the capacity of osmotic adjustment, but also be an indicator for measuring plant metabolism. The contents of total proteins in the root of *Kandelia candel* treated under salt stress for different time were determined, which showed that the contents of total proteins in the root of *Kandelia candel* were reduced with the increasing concentrations of NaCl and were lower than that of the control. The contents of total proteins in three treatments (150, 300 and 450 mmol/L) were decreased by 5.9%, 20.6% and 27.4%, respectively, compared with the control at 3 h. The contents of total proteins in the root of *Kandelia candel* were gradually reduced with the increase of processing time within 24 h. At 24 h of processing, the contents of total proteins in the root of *Kandelia candel* treated with low concentrations of NaCl (150 and 300 mmol/L) showed no significant differences as compared with the control, while that in the root of *Kandelia candel* treated with 450 mmol/L of NaCl was significantly lower than that of the control (P <0.01), which indicated that the salt stress had affecdated the synthesis of total proteins in the root of *Kandelia cande* (Figure 1).

### Effects of salt stress on the antioxint defense system

#### Effects of salt stress on the activities of CAT

**Figure 2:**
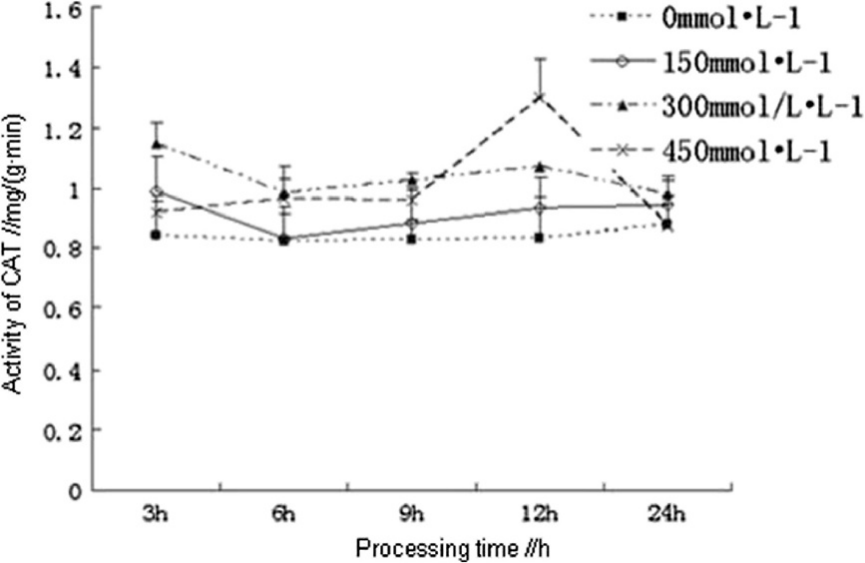
**Effects of salt stress on the activity of CAT in the root of** ***Kandelia candel.***

Catalase (CAT) could decompose hydrogen peroxide into water and oxygen to remove the peroxide in plants, and the higher activity of CAT was, the stronger salt tolerance would be (Ho et al. [1998]). The activities of CAT in the root of *Kandelia candel* treated under stress were determined, which showed that the activities of CAT were gradually increased with the increasing concentrations of NaCl and were higher than that of the control, although there were some slight differences among different concentrations in different processing time. The activities of CAT in each treatment showed a gradually ascent trend after a slightly declined within 12 h. At 12 h of processing, activities of CAT in each treatments had reached the maximum, particularly the activity of CAT in the root of *Kandelia candel* treated with 450 mmol/L of NaCl was significantly higher (P <0.05) than that of other treatments, which had increased by 56.42%, compared with the control, and the differences were at a significant level (P <0.01). After 12 h, activities of CAT in each treatment were all reduced (Figure 2). At 24 hour, the activities of CAT in the root of *Kandelia candel* treated with three concentrations of NaCl were all close to the control. As can be seen that low concentrations of NaCl showed little effect on the activities of CAT in the root of *Kandelia candel* which were not significantly different in processing time; while *Kandelia candel* treated with high concentration of NaCl (450 mmol/L) could activate CAT activity, thereby reducing the harm caused by oxidative stress, *Kandelia candel* was adapted to the salt stress environment after 24 h, so the activities of CAT had decreased to the control levels.

### Effects of salt stress on the activity of POD

**Figure 3:**
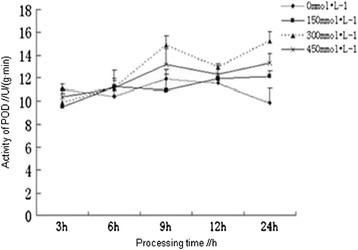
**Effects of salt stress on the activity of POD in the root of** ***Kandelia candel.***

Peroxidase (POD) could remove H_2_O_2_ in plants to prevent the cell membrane from oxidation by H_2_O_2_. Therefore, the enhancement of POD activity could effectively resist the oxidative stress caused by salt stress, thereby improving the salt tolerance of plants (Mandhania et al. [2006]). Different concentrations (150, 300 and 450 mmol /L) of NaCl solution were used for the processing of *Kandelia candel* seedlings, the POD activity had shown an overall increasing trend with the increase of processing time, which had reached the maximum (12.09, 15.19 and 13.32 u/g.min^−1^) in each treatment at 24 h and were all significantly higher than that of the control (10.45 u/g.min^−1^) (P <0.05). At the early stage of processing (≤6 h), the activities of POD in each treatment were not significantly different, which were even lower than that of the control. After 6 h, the activities of POD in each treatment had gradually increased with the increasing concentrations of NaCl (Figure 3). The POD activity of the roots treated with 300 mmol/L of NaCl was significantly higher than that of the other treatments during 6–24 h, it was to be further studied that whether this result was a performance of the adaption of mangrove plants to medium salinity environment.

### Effects of salt stress on the activity of SOD

**Figure 4:**
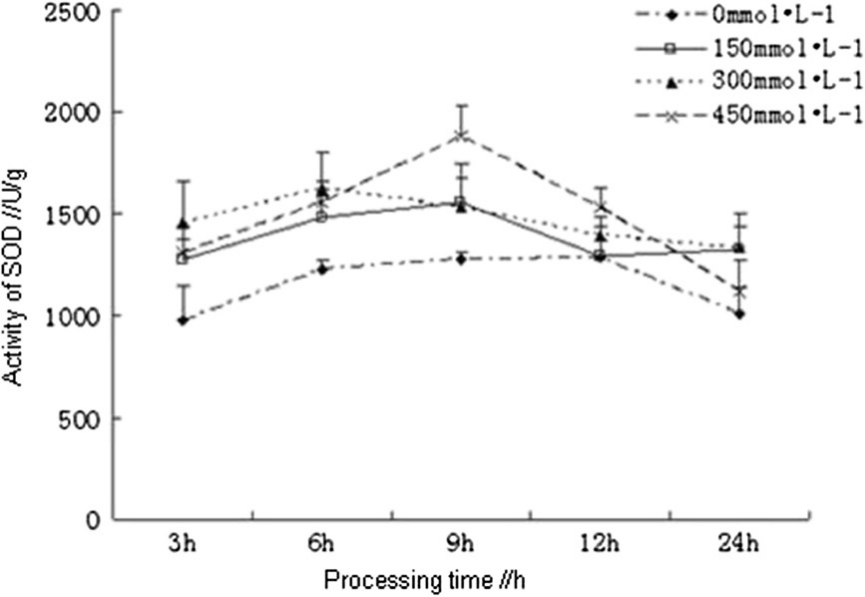
**Effects of salt stress on the activity of SOD in the root of** ***Kandelia candel.***

Superoxide dismutase (SOD) could catalyze disproportionation reaction of two superoxide radicals to generate O_2_ and H_2_O_2_, and then the H_2_O_2_ was catalyzed and removed by POD and CAT, which was the response started first in the resistance to oxidative stress of plants (Cheruth et al. [2009]). The activities of SOD in the root of *Kandelia candel* treated with different concentrations of NaCl had shown a trend from ascent to descent, which had reached the maximum at 9 h and were reduced to the initial level at 24 h. At the early stage of processing (≤6 h), the activities of SOD in the root of *Kandelia candel* were significantly higher than that of the control and were not significantly different among each treatment (Figure 4). After 6 h, high concentration (450 mmol/L) of NaCl had caused a sharp increase of SOD activity, while the activity of SOD in the root of *Kandelia candel* treated with low concentration of NaCl changed slowly, especially in 300 mmol/L of NaCl solution, which might be a performance of the adaption of mangrove plants to medium salinity environment as well.

### Effects of salt stress on the content of ASA

**Figure 5:**
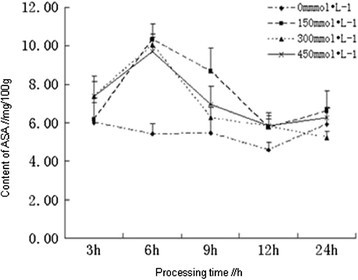
**Effects of salt stress on the content of ASA in the root of** ***Kandelia candel.***

As an active oxygen scavenger, Ascorbic acid (ASA) could clear the damages caused by superoxide radicals (O_2_^−^), singlet oxygen (^1^O_2_), hydrogen peroxide (H_2_O_2_) and other active oxygen, and played important roles in defense of plants against the toxic effect of active oxygen (Cheruth et al. [2009]). The contents of ASA in the root of *Kandelia candel* treated with different concentrations of NaCl were determined, which indicated that salt stress with different concentrations could all cause the accumulation of ASA, and the contents were higher than the control. The contents of ASA in the root of *Kandelia candel* in each treatment had shown a trend from ascent to descent within 24 h. At the early stage of processing (≤6 h), the contents of ASA had increased dramatically, which had reached the maximum (10.2998, 10.0496 and 9.7234 mg/100 g respectively) in each treatment (150, 300 and 450 mmol/L) at 6 h, but the differences were not significant among different concentrations of NaCl. Subsequently, the contents of ASA were gradually reduced, which had decreased to the untreated levels in each treatment at 24 h and were not significantly compared with the control (Figure 5). During the entire process, there was no significant difference among different concentrations of NaCl, which indicated that *Kandelia candel* first activated the ASA reaction to reduce the damages of free oxygen radicals to plants both under high-salt and low-salt stress. With the increase of salt concentration or processing time, when the contents of ASA were unable to meet the demands, *Kandelia candel* activated antioxidant enzyme system (CAT, POD and SOD) to further enhance the antioxidant defense capacity.

### Effects of salt stress on the content of MDA

**Figure 6:**
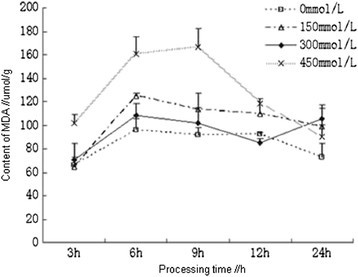
**Effects of salt stress on the content of MDA in the root of** ***Kandelia candel.***

Variation of malondialdehyde (MDA) content was an important indicator to measure lipid peroxidation, the higher the MDA content was, the higher the oxidation degree of plant cell membranes and the greater of damage would be (Cheruth et al. [2009]). The contents of MDA in the root of *Kandelia candel* treated with different concentrations of NaCl had shown an overall trend from ascent to descent. At the early stage of processing (≤6 h), the contents of MDA in each treatment had increased dramatically with the increasing concentrations of NaCl, which had reached the maximum at 6 h; subsequently, the contents of MDA in low concentrations of NaCl solution were slowly reduced, while that had declined dramatically in high concentrations of NaCl solution had declined dramatically after a brief period of stability; after 12 h, the contents of MDA in each treatment were not significantly different compared with the control. This suggested that the salt stress-induced lipid peroxidation had been basically cleared. However, the contents of MDA in moderate concentrations (150 and 300 mmol/L) of NaCl solution in each processing time were not significantly different compared with the control, which had further confirmed the adaptability of *Kandelia candel* to medium salinity environment. In 450 mmol/L of NaCl solution, the root of *Kandelia candel* was rapidly damaged by lipid peroxidation, and the contents of MDA had reached the maximum level at 6 h (no significant differences were observed at 6 h and 9 h), while the antioxidant defense system in plants had cleared the oxygen free radicals generated by salt stress, thus reducing the lipid peroxidation of cell membrane to the normal levels (Figure 6).

## Discussion

As a normal physiological response, plants generated oxidative stress immediately when under salt stress, which led to the membrane lipid peroxidation, result in the dramatically increasing contents of MDA, and the emergence of this signal could induce the activation of antioxidant defense system and the expression of related enzymes in plants (Liang et al. [2008]). However, before the expression of these enzymes had reached the peak, plants had to activate ASA for protection and to clear part of the active oxygen, so the ASA contents had attained the maximum within 6 h simultaneously; when all the antioxidant-related enzymes in plants were expressed, these enzymes had played major roles instead of ASA, so the ASA contents were reduced after 6 h; with the increasing activities, these enzymes had taken charge essentially at 9 h, and the contents of ASA had declined to near control levels, the contents of MDA were reduced more significantly. Therefore, in the process of resistance to oxidative stress, plants first activated the ASA pathway, but the antioxidant enzyme system had played key roles.

Among the enzymes we had determined, plants first activated SOD, which catalyzed disproportionation reaction of two superoxide radicals to generate O_2_ and H_2_O_2_, resulting in the ascent trend of SOD in plants within 9 h, in the meantime, POD was required to clear the H_2_O_2_ and O_2_, products from SOD pathway, resulting in the simultaneous increase of POD. The activities of CAT had shown a trend from descent to ascent, which might indicate that, the main function of CAT was to remove H_2_O_2_. However, other peroxides but not H_2_O_2_ were first observed in the oxidative stress of mangrove plants, so the demand for POD was earlier than for CAT, with the accumulation of H_2_O_2_ in cells at the late stage, CAT was required to clear the H_2_O_2_, resulting in the peak of CAT activities at 12 h.

Antioxidant defense system in plants was composed of a number of enzymes and antioxidant substances which could remove the active oxygen, including superoxide dismutase (SOD), peroxidase (POD), catalase (CAT) and ascorbic acid (ASA), by their synergistic effects plants were able to resist the salt stress-induced oxidative damages. SOD was the most important antioxidant enzymes in the entire antioxidant defense system (Peng [2005]). In addition, ASA and some other antioxidant substances in plants had physiological functions to remove free radicals (Liu et al. [1997]). Increasing activities of antioxidant enzymes and improving antioxidant metabolism in plants was one of the most important ways to enhance salt tolerance of plants (Mao et al. [2004]). In this study, different concentrations of NaCl solution were used for salt stress treatment on *Kandelia candel* with different processing time, the results showed that the contents of total protein in the root of *Kandelia candel* were reduced with the increasing concentrations of NaCl with the same processing time, but the contents of total protein in the root of *Kandelia candel* treated with low and moderate concentrations of NaCl were decreasingly varied from the control with the increasing processing time. However, Ma *et al.* had reported that the contents of protein in the radicle of *Kandelia candel* under salt stress for 30 d had increased with the increasing salinity (NaCl concentration is greater than 20‰) (Ma et al. [2002]), which was not consistent with the results of this study and might be resulting from the different processing time under salt stress, because the salt resistance of halophytes was related to the duration of salt stress (Liu and Zhang [1994]). The activities of SOD and CAT in the root of *Kandelia candel* treated with high concentration (450 mmol/L) of NaCl had reached the maximum at 9 h and 12 h, respectively. In moderate concentrations of NaCl solution, activities of CAT, POD and SOD in the root of *Kandelia candel* were basically the highest. When treated for 24 h, the activities of SOD had shown a trend from ascent to descent with the increasing concentrations of NaCl, which was consistent with results of the research on SOD activity in leaves of *Kandelia candel* under short-term salt stress by Wang et al. (Wang and Lin [2000]).

Previous research had shown that low temperature, drought, high salt, strong radiation and other conditions aggravated the membrane lipid peroxidation in plants, and MDA contents was an important indicator to measure lipid peroxidation (Zheng and Lin [1998]). It could be seen from the results of our study that, the contents of MDA in the root of *Kandelia candel* had increased in the initial 9 h of salt stress, which were reduced after 12 h and were even lower than that of the control in 300 mmol/L of NaCl solution. Enzymes removing active oxygen (CAT, POD and SOD) had basically attained the maximum activities in the first 12 h of salt stress, which can be inferred that at the early stage of salt stress, the membrane lipid peroxidation was aggravated, the contents of MDA had increased, and the activities of antioxidant enzymes were enhanced to remove active oxygen, thereby reducing the contents of MDA in the root of *Kandelia candel*. Consequently, the decrease of MDA contents was later than the achievement of the maximum activities of antioxidant enzymes (CAT, POD and SOD), which was consistent with results of this research. In 300 mmol/L of NaCl solution, enzymes and antioxidant substances (CAT, POD, SOD and ASA) which could remove the active oxygen in the root of *Kandelia candel* had first reached the maximum, and the contents of MDA were the minimum compared with that in the other two treatments, indicating that the protection of antioxidant defense system in the root of *Kandelia candel* treated with 300 mmol/L of NaCl was better than that in the other two treatments.

## Conclusions

In this study, the SOD, CAT and POD activities and contents of soluble protein, ASA and MDA were measured in the seedling roots of *Kandelia candel* treated with different concentrations of salt for different processing time. The results showed that a certain salt concentration (<300 mmol/L) was required for the growth of *Kandelia candel* seedlings. At the early stage of high-salt stress, *Kandelia candel* can rapidly activate antioxidant defense system to resist the salt induced oxidative stress, thus reducing the damages of oxidative stress to plasma membrane, which might be an effective means for *Kandelia candel* to resist high salt stress. This may provide a theoretical basis for further analysis on the physiological mechanism of salt tolerance of mangrove plants.

## Authors’ original submitted files for images

Below are the links to the authors’ original submitted files for images. Authors’ original file for figure 1 Authors’ original file for figure 2 Authors’ original file for figure 3 Authors’ original file for figure 4 Authors’ original file for figure 5 Authors’ original file for figure 6
