# Supplementary figures and images for: Effects of salt stress on antioxidant defense system in the root of Kandelia candel
Source: Bot Stud. 2014 Jul 23;55:57. doi: 10.1186/s40529-014-0057-3 (PMC5430347; doi:10.1186/s40529-014-0057-3)

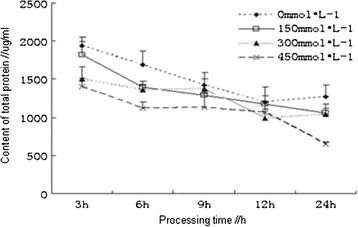

Supplement: Supplementary file 1 — Authors’ original file for figure 1 [file 40529_2014_9057_MOESM1_ESM.gif]

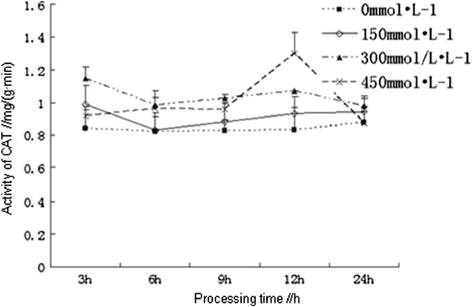

Supplement: Supplementary file 2 — Authors’ original file for figure 2 [file 40529_2014_9057_MOESM2_ESM.gif]

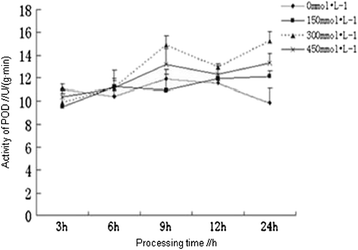

Supplement: Supplementary file 3 — Authors’ original file for figure 3 [file 40529_2014_9057_MOESM3_ESM.gif]

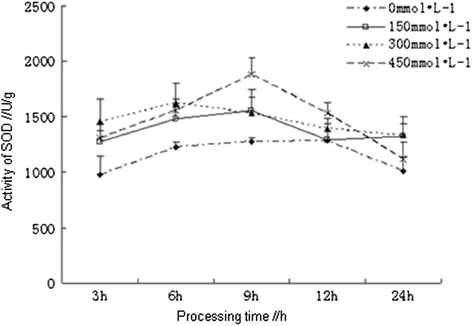

Supplement: Supplementary file 4 — Authors’ original file for figure 4 [file 40529_2014_9057_MOESM4_ESM.gif]

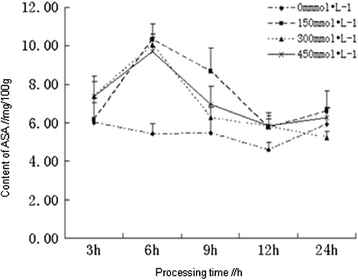

Supplement: Supplementary file 5 — Authors’ original file for figure 5 [file 40529_2014_9057_MOESM5_ESM.gif]

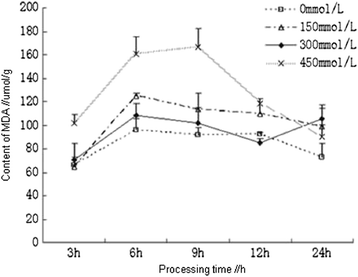

Supplement: Supplementary file 6 — Authors’ original file for figure 6 [file 40529_2014_9057_MOESM6_ESM.gif]
